# Irregularities of the Teeth and Their Treatment

**Published:** 1901-11

**Authors:** 


### Bibliography.

Irregularities of the Teeth and their Treatment. By Eugene S. Talbot, M.D., D.D.S., Professor of Dental and Oral Surgery, Northwestern University, Women's Medical School, Honorary President of the Dental Section of the Tenth International Medical Congress, Berlin, 1890, etc., etc. Fourth Edition, with five hundred and eighty Illustrations. F. A. Davis Company, Publishers, Philadelphia, 1901.

This, the fourth, edition of Dr. Talbot's well-known work on Irregularities of the Teeth has been rewritten and rearranged with plenty of new maternal and new facts," so that it may be regarded practically as a new work.

If we except Dr. Farrar's great work upon the same subject, no author has undertaken to approach this subject from the foundation studies of its etiology, and no one, so far as the reviewer is aware, has attempted to consider this subject from the stand-point of the author. In his preface he writes: "The unwritten law in general medicine, 'that to know the cause is half the treatment,' is also applicable in the treatment of deformed jaws and irregularities of the teeth. . . . Without the knowledge of etiology no one can successfully correct deformities, as is evident in the many failures by men who profess to make this a specialty. . . . He has confined himself entirely to his own appliances and methods of treatment. 'Systems,' in the ordinary Charlatan-like sense, are not accepted as guides. In the author's opinion the practitioner should be familiar with the etiology of the case in hand; his knowledge of principles and mechanics should suggest to him the most suitable appliances for the given case."

This quotation is sufficiently clear that the author has no sympathy with methods which have come into quite general use in the regulation of teeth, and this is made very evident as the reader proceeds in the examination of the various chapters. Whether this opposition to "systems" is based on good grounds, it must be conceded that the author's undisguised opposition to this method is correct if they are made use of without a previous study of the etiology of the subject, but with this intelligence it is difficult to understand why these cannot be used as effectively as an appliance planned for a given case.

In order that our readers may have some idea of the scope of this volume a portion of the " Contents" is given. Seventeen pages are devoted to History, followed by Heredity, Congenital Factors and Maternal Impressions, Post-Natal Skull and Jaw Development and Periods of Stress, Development of the Cranium and Face, Development of the Jaws, Development of the Alveolar Process, Development of the Vault, Development of the Peridental Membrane, Development of the Teeth, Social Consanguinity, Near-Kin, Early and Late Marriage, etc. The practical subject, which includes appliances, is not reached until the last chapter, under the head of " Surgical Corrections," and this covers forty-four pages. It will thus be seen that the author has not regarded as of much importance the mere description of cases or the preparation of appliances used.

While this may be to some a decided defect in the book, and in the reviewer's opinion it is not satisfactory, it must be remembered that the author's aim is evidently not to write for the mere mechanic, but for the man who is searching for the reason why these irregularities should occur; and if this reason be known, the appliance necessary for its correction is a secondary consideration.

Whether the reader will agree with the author or not in his general argument and his marshalling of facts to support it, it must be acknowledged that he has produced not only an interesting book, but one full of facts that go far to sustain his conclusions.

In the chapter on the " Development of the Jaws, this curious fact is stated presumably on exact measurements. " The lateral diameter of jaws of existent races in Europe is greater than of the same races in America. . . . The jaws in older parts of the United States are smaller than jaws in the newer. The differences between jaws of the residents of Boston and those of Chicago are thus in evidence." Presumably it is to be inferred that the farther west the child is born there will be a proportionate increase in the diameter of the jaw; a discouraging fact, if it be a fact, for those born on the Pacific coast, as they will rapidly develop into the large jaws of the American Indian.

The author discards " dome," " palate," and " arch," in speaking of the roof of the mouth and adopts the word " vault." This includes the hard palate, the soft palate, and the alveolar process.

The author says, "The height of the vault in most cases is far below the average of the present day. In four thousand six hundred and fourteen measurements of normal individuals, made by the author, it was found that the average height of the arch was 0.58 of an inch. The measurement was made from the alveolar border between the second bicuspid and the first permanent molar to the height of the arch. The average of two hundred and fifty-one skulls of ancient and modern Romans, Indians, etc., was 0.56." He further says, "Narrow jaws are rarely observed among pure races. In the examination of the thousands of skulls of early races in the museums of Europe and this country, very few, if any, contracted vaults are found."

Those who have been made familiar with the author's views in his previous work on "Degeneracy: Its Causes, Signs, and Results," will recognize much of that valuable work, in substance at least, in many of the chapters.

Very few, it is surmised, will be prepared to endorse the following relating to consanguineous unions. "The error of the old doctrine upon which was founded the prohibition of consanguineous unions lay, as Strahan remarks, not in asserting that disease and deformity were more often met with in children of these than of other unions, for such is the fact, but in attributing these unhappy results merely to parental blood kindred. Over and above the fact that these consanguineous marriages are almost certain to transmit, in an accentuated form, defect or tendency to disease already present in the family, there is no physiologic reason why such marriages should not take place." This seems to the reviewer to be assuming too much on insufficient data. The fact remains that our asylums are full of the results of such marriages, and the evidence seems to lean strongly in the direction of the truth of the "old doctrine."

These various chapters are all of them intensely interesting to the searcher after the hidden causes of disease, yet there is much in all of these that seems to bear but a remote relation to the etiology of irregularities. Take, for instance, the chapter on "Intellectual and Moral Defects." Taken independently, it is full of valuable material, but it is difficult to see what intimate connection the "moral imbecile" or the paranoiac has with changes leading up to malposition of the teeth, and the pages spent in delineating their peculiarities.

With Chapter XVII., on "Developmental Neuroses of the Face," the author begins the more direct study of matter leading directly up to irregularities, and no criticism can justly be made of the very able marshalling of facts as the reader proceeds. Developmental Neuroses of the Face is followed by The Nose and Interior Facial Bones,—of the eye; bones of the ear; jaws of the seemingly normal; of the maxillary bones; of the vault; of the palate; in teeth position, and this through many pages, covering much of vital importance in the etiology of the subject, but of which space will not permit extended quotations.

"Surgical diagnoses" is the beginning of treatment. It is satisfactory to note, under this heading, that the author has a limit in age in which regulating can be safely performed. There has been a growing tendency to regard it as possible at any age short of senility. The physiological and pathological reasons why this is a mistake is clearly set forth in the following quotation: "The chances for perfectly satisfactory results in regulating decrease yearly after puberty, and after twenty-six are very meagre. At this time the osseous system is fully developed. An unusual amount of force is required to set up inflammation and absorption. It is possible to regulate deformities as late as the thirtieth year. The resulting pain is, however, so severe and the mechanical force necessary to produce absorption of the obstructive portions of the alveoli so great, that the results hardly justify the procedure."

The author's description of the appliances in use in his practice will hardly be regarded as satisfactory. The practical mind will naturally ask, Why confine these to his own experience? His answer is, as given, that they have served his purpose. The reviewer's opinion is that, if much of the matter published in previous works of the author had been eliminated and in its place a more detailed description of appliances and how to make these, the book would have been more valuable to the average dental mind. To one, however, who does not need these practical suggestions the book will be full of matter for thought, and it is for these the author has prepared the volume.

The work of the publisher is deserving of unstinted praise. Everything connected with the book is of a high order of excellence.

